# 微课在胸腔外科临床教学中的应用

**DOI:** 10.3779/j.issn.1009-3419.2018.04.02

**Published:** 2018-04-20

**Authors:** 小飞 李, 杰 雷

**Affiliations:** 710038 西安，第四军医大学唐都医院胸腔外科 Department of Thoracic Surgery, Tangdu Hospital, Fourth Military Medical University, Xi'an 710038, China

**Keywords:** 微课, 教学模式, 胸腔外科, Micro-lecture, Model of teaching, Thoracic surgery

## Abstract

当前，随着网络信息技术的飞速发展，微课这一新的教学模式在现代教学活动中发挥越来越重要的作用，其短小精悍的教学内容能快捷、方便、反复获取，提高了学生学习效率和自主学习能力。胸腔外科临床教学尚处于摸索发展阶段，需要不断深化改革，引入适应社会发展且适合学员学习特点的新的教学模式以提升临床教学效果。本文就“微课”的概念、特点及优势进行阐述，同时对其在胸腔外科教学过程中应用进行探讨和思考，以进一步促进微课在胸腔外科临床教学中合理应用。

## Abstract (en)

Today, with the rapid development of network information technology, the micro-lecture plays a role in the teaching activities is becoming more and more important. The short and efficient teaching content of micro-lecture can be downloaded rapidly, expediently, and repeatedly, which improve the learning efficiency and independent learning capability. The clinical training of thoracic surgery elementarily remains at the scrabble stage. We require continuous reform and introduce new modes of teaching, which compatible with the development of society and the study habits of novice, to enhance the effectiveness of clinical training. In this paper, the concept, characteristic and advantage of micro-lecture was discussed, and the feasibility of application of micro-lecture in thoracic surgery teaching was also discussed. Our aim was to promote the application of micro-lecture in the clinical training of thoracic surgery reasonable and extensive.

胸腔外科疾病发病率高，临床表现多样，有的疾病进展迅速，需要最短时间做出正确的诊断、采取最恰当的治疗措施才能最大程度挽救患者性命，这就要求每一个胸腔外科的从业者或学员必须具备良好的心理素质和丰富的专业知识，也就势必要求我们的临床教学必须提供足够深、足够新、足够广的教学内容^[1]^。基于胸腔外科知识点多且杂，手术操作风险高的特点，我们必须对教学模式进行创新，应用更为快捷获取，更为方便查阅，随时、随地能够学习的学习模式融入胸腔外科临床教学。随着网络平台及智能终端的飞速发展，以移动通讯和教学资源共享为依托的网络教学模式已逐渐应用于现代教学，微课就是其中一种。微课以时间短、内容精、形式新为主要特征，符合学员的学习习惯，从一开始就备受欢迎，逐渐成为网络社会的热点^[2, 3]^。通过5 min-10 min的小视频展示，针对某一知识点进行直观详细的讲解，借助网络平台的扩散，存储于智能终端，有针对性的反复下载、学习，微课有助于提高学习的自主性、目的性，非常契合胸腔外科从业者学习特性，因此，将微课教学融入胸腔外科临床教学具有良好的应用前景。

## 1 微课的概念及特点

微课是当前“微”教学模式的典型代表，这一概念由美国新墨西哥州圣胡安学院的高级教学设计师David Penrose于2008年首先提出。其定义是指使用" 微视频" 作为记录，时间多在10 min以内，有明确教学目标，内容短小，集中说明问题的小课程，综合使用了文本、图像、表格、音频、视频和动画等多种资源；其核心在于：以学生为中心，根据学生的需求，提供便捷的面向学生的学习内容。随着网络通讯的发展和智能手机、电脑、iPad的普及，学生自主学习已经成为可能，教师把学习中的重点和难点、热点等制作成微课，上传到网上，学生便可以随时点播学习从而提高学生的自主学习能力^[4, 5]^。

目前，微课经过不断的发展和完善，形成了自身的特点^[6, 7]^。①短小精悍。微课针对某一特定知识点进行教学，目标明确，主题突出，时间短、内容精。张力性气胸微课针对张力性气胸产生的原因、患者的临床表现及诊治措施进行讲解，有助于学员快速了解这一疾病的特征；②直观形象。微课借助短视频进行展示，综合应用了3D打印模型、动漫、图片等直观的形式进行展现。胸腔闭式引流术微课应用操作过程的视频进行讲解，将操作步骤及注意事项融入其中，有助于学员尽快了解掌握；③方便快捷。微课借助网络平台进行发布，使用智能终端进行下载、学习，学员可根据需要随时、随地、反复下载、学习。肺癌肿瘤-淋巴结-转移（tumor-node-metastasis, TNM）分期微课就肺癌TNM分期各个分期标准进行讲解，存储于iPad、手机等智能终端，随时、随地翻阅、学习，有助于学员加深记忆。

## 2 微课在胸腔外科教学中的优势

目前，胸腔外科教学多采用基于课堂的传统教学方法，以授大课的形式向学员讲授相关知识，其优点是知识框架完整，信息量大，教师易于根据课程标准进行授课，为我国培养了大批优秀的胸腔外科医师。但是，这种方法存在一定的缺陷^[8]^。①课堂时间较长，信息量大。研究已经证实，人最佳的专注时间大约为10 min，超过这一时间段则注意力大大下降，对知识的理解吸收减少；②时间固定，时效性差。课堂授课，集中于教室进行，多在固定时间进行，但是人的学习兴奋时间点不同，许多学员正课时间犯困，学习效果差；③复习手段受限，遗忘率高。课堂授课结束后，学员复习则主要依靠书本或授课幻灯复习，无法从“冰冷的”文字中了解更多的相关知识，而且随着时间的延长，复习效果越差，遗忘的重要知识点越多。而微课这一新的教学模式能够很好的弥补上述缺点，可视为现行教学模式的最佳补充，展示独特的教学优势^[9-11]^。

### 2.1 创造情景化场景

微课把要阐述的知识点控制在10 min左右，学生更易于集中精神，并且微课一般会根据讲授内容营造情景化场景，有助于加深记忆。以胸腔外科常见的“气胸的诊断”为例，在给患者行视、触、叩、听等体格检查时，视频显示授课医师给患者检查操作的过程，配合视频中的讲解及患者的表现，学生很容易记住体格检查对于气胸诊断的重要性及操作规范。

### 2.2 增加学习兴趣，提高实践能力

相比语言文字传授，通过视频学习更能激发学生的兴趣^[12]^。比如胸腔外科最常采用的诊断和治疗技术之一胸腔闭式引流术，它是临床医生及医学生必须掌握的基本技能之一。通过微课以视频的形式让医学生学习胸腔闭式引流术的操作规范，观看教员对真实病人的实际操作，不仅增加了学生的学习兴趣，并且在操作过程中，操作者会将重点、难点及注意事项给予强调或近距离拍摄。学生再结合理论知识的学习，间接提高了实践能力，减少了实际操作时的恐惧。

### 2.3 使学习时间更加自由化

随着网络终端设备的普及，智能手机甚至平板电脑在大学生中使用率非常高。将微课资源下载到手机或电脑终端，针对课堂中没有理解或掌握的概念、操作等，可以在任何能够使用手机的地方学习，并且可以重复播放。例如针对胸外科肺癌患者TNM分期的学习。由于其数字较多，且分类复杂，在课堂上很难完全掌握。课堂之下通过下载相关微课资源包，结合视频中有关TNM分期的影像学及解剖学图片，能更方便的掌握肺癌的TNM分期。

### 2.4 促进了教学资源的模块化

整合微课可以视作一种模块化的整体。将微课中的教学方案、教学设计、教学内容、课堂教学时使用到的多媒体素材和课件、教师课后的教学反思、学生反馈意见及教学专家的点评意见等相关教学资源，构成了一个主题明确、类型多样、结构完整的教学资源包。同时微课通过将教学资源与教学任务、教学活动、教学环境之间建立有意义的关联，营造出一个完整的结构化资源应用环境，从而在提高教师的业务水平及学生的学习质量^[13]^。

## 3 微课在胸腔外科教学中的应用

临床胸腔外科疾病学习中，我们需对疾病的概念、解剖学结构、病理生理改变、疾病的分类、诊断、鉴别诊断及救治等进行系统深入的认识。由此可见，临床每一个疾病的知识点都十分繁琐和抽象，学习起来比较枯燥。微课教学的应用就应该重点、难点、热点突出，分而治之；各个流程相互衔接，内容精细、目的明确，力求最短时间呈现最精彩的内容。

① 通过问卷调查法、直接观察法等研究方法了解各阶段学员胸腔外科学习中的重点、难点及热点，根据这“三点”确立微课内容。

② 采用动漫、视频、3D模型、Flash、PPT等多种直观形式呈现微课内容并反复改进，精化、优化内容细节，并针对不同疾病形成资源有序、内容完整的教学资源包，将疾病的众多知识点分别进行独立而细致的阐释。

③ 借助网络平台将微课内容发布于智能终端供学员下载、学习、复习，在临床工作中进行不断的强化。

④ 最终通过闭卷考试及实践操作总结微课教学的优点与不足，进而逐渐将微课教学系统地应用于胸腔外科教学中，形成微课教学方案。

我们以胸腔外科最常见的疾病“气胸”为例，探讨微课教学在胸外科教学中的应用。气胸作为胸外科最常见的疾病之一，临床医生和医学生应当从胸膜腔解剖结构、病理生理改变、分类等方面对气胸进行深入认识，能够对气胸进行明确的诊断、鉴别诊断和治疗，必要时还需要进行快速的现场急救。为此我们把以上几方面内容通过视频、3D模型、PPT等形式分别制作成微课，课件中穿插图片、三维动画、相关场景及操作视频等，形成" 气胸" 的教学资源包。再将教学资源包分享给胸外科临床实习生、进修生、规范化培训住院医师等，学员可通过手机、平板电脑、MP4等下载“气胸”的教学资源包，随时学习。有利于新学员在进行首次接诊或首次操作时进行突击学习，更好的做好医患沟通或操作步骤，进而使其在学习过程中更加主动，兴趣更高。例如，我们利用3D模型和三维动画的形式分别呈现出胸膜腔解剖结构及病理生理改变。学生通过这种方式学习后，胸膜腔的解剖结构以及气胸时胸膜腔的病理生理变化就在其脑海中有一个生动的画面，遇到气胸病人时，能够更形象地理解其解剖结构改变。再例如，我们将胸腔穿刺术的操作步骤及操作注意事项以3D动漫及实操视频的形式讲解操作过程，穿刺前的准备、穿刺的进针点、穿刺中的流程、穿刺针位置的判断方法、穿刺后的观察等细节内容逐一直观展现，使得学生在操作过程中能够明确的了解操作方法，既有助于增强操作信心，又有助于提高操作水平。

最后，我们以闭卷理论考试、临床实践操作以及平时互动问答表现等三方面对微课内容及学员学习效果进行量化考核。通过对多批次学员综合量化考核，一方面比较微课教学的优点与不足，不断改进微课内容，精益求精；一方面测试学习成绩，了解学员通过微课教学对理论学习能力、实践操作能力的提升程度，客观实际，最终形成贴近胸外科临床教学的教学方案。

## 4 微课在胸外科教学应用中的思考

### 4.1 合理选题

微课在胸外科教学应用中应合理选题在制作微课视频时应聚焦于临床教学过程中的重点、难点及热点，不能选题过大^[14]^。微课程本身短小、精悍，时间一般限制在5 min-10 min，当面对较复杂的知识体系时，其学习效果就会逊于具有较大容量的学科教学这一方式。因此，对于胸外科中结构简单，易于通过短小视频阐明的知识点可以合理的应用。但对于内容结构复杂的知识，不宜为了制作微课而将该知识点过度划分，使划分后的知识点缺乏连贯性，成为知识孤岛。

### 4.2 课件设计

微课在课件设计过程中应特点鲜明、结构合理如果制作的微课不能将知识点的重点、难点及热点突出，而仅仅将课堂的讲课过程通过录像的形式分批次展示出来，使学生只停留在浏览、知晓的肤浅层面，学生不能很好的从中汲取精华^[15, 16]^。微课视频制作时，也应注意结构的合理性。如片头、片尾过长，为追求上课的形式而将上课起立、师生互相问候等录制过多，就会轻重不分。课件制作时，还可能出现教师出镜过多而分散学生注意力，过度追求华丽、趣味而使学生将注意力从内容转移到形式上，有些课件反而是缺乏情景设计，不能激发学生的兴趣。

### 4.3 整合资源

设计制作微课程需要更多资源的整合教师在设计制作微课程之前，首先要对微课有全面的认识，理解微课的核心本质、教育价值、应用优势及发展趋势，其次要广泛学习微课的创作方法和制作技术^[17]^。通过观摩全国微课大赛获奖作品，从获奖作品的选题、设计、内容及技术等几方面借鉴其优点。然后根据自身教学过程中的重点、热点及学生反映的学习难点、疑点合理设计微课内容及表现手法，同时还要加强同类学科之间的交流以及增强相关科室之间的协作。最终还需要科室团队的智慧和力量将整合的所有资源整合成为微课程教学资源包。

因此，作为网络社会“微时代”背景下的一个新的教学模式，微课这一理念还有待于进一步界定和完善，内容建设还需要更加系统化和规范化，其在胸腔外科临床教学过程中的应用也需依赖于研究者、教师、学员更深入的研究和更广泛的实践。胸腔外科是一个不断更新，不断发展的学科，微课在胸腔外科教学中的应用也必定是一个持续变化完善的过程，精雕细琢，最终服务于我们胸腔外科的每一个从业者，提升整个行业的综合素质。
